# 纵隔肿物活检小标本的获取与病理报告指南

**DOI:** 10.3779/j.issn.1009-3419.2014.02.07

**Published:** 2014-02-20

**Authors:** Alberto Marchevsky, Alex Marx, Philipp Strobel, Saul Suster, Federico Venuta, Mirella Marino, Samuel Yousem, Maureen Zakowski, 北京大学肿瘤医院 付浩

**Affiliations:** 1 Department of Pathology and Laboratory Medicine, Cedars Sinai Medical Center, Los Angeles, California; 2 Department of Pathology, University Medical Center Mannheim, Mannheim, Germany; 3 Department of Pathology, University Medical Center Mannheim, Mannheim, Germany; 4 Department of Pathology and Laboratory Medicine, Medical College of Wisconsin, Mil-waukee, Wisconsin; 5 Department of Toracic Surgery, University of Rome La Sapienza, Rome, Italy; 6 Department of Pathology, Regina Elena National Cancer Institute, Rome, Italy; 7 Department of Pathology, UPMCPresbyterian University Hospital, Pitsburgh, Pennsylvania; 8 Department of Pathology, Memorial Sloan Ketering Cancer Center, New York

目前，胸腺恶性肿瘤治疗方案大多是根据术后病理确定，然而，多数临床治疗决策需要在术前通过活检小标本的病理报告来制定。所以，术前活检小标本的正确获取和病理解读对治疗决策的制定显得非常重要^[1]^。这些标本包括细针活检标本，带芯穿刺活检标本和手术切取活检标本^[2-7]^。由于胸腺肿瘤的病理诊断对组织的获取方法和获取量都有较高的要求，加之对病理的描述也没有统一的标准，使得小标本在诊断胸腺瘤方面存在诸多问题。为此，ITMIG在病理科医生和外科医生回顾相关文献和提出初步建议的基础上，经集体讨论制定了活检规范操作流程，提出了对纵隔肿物小活检标本处理和病理报告的建议。旨在为术前患者的治疗提供一个统一和具有循证医学证据的方法；同时，将有利于全球数据之间的比较和开展合作研究，充分利用医疗资源。

## 1 纵隔肿物活检方法的概述

纵隔肿瘤、囊肿或其它病变的病理标本获取技术有X线透视、胸部超声或CT引导下穿刺，胸腔镜手术，前纵隔切开术和小切口开胸术或Chamberlain手术^[1-7]^。总的来说均较安全，发生气胸、出血或其他并发症的概率不到5%。但有报道认为肺门处肿物经胸穿刺活检发生气胸的概率高达34%。纵隔囊肿活检偶有感染的报道。活检后种植转移仅限于极少数的个案报道^[8, 9]^。位于前纵隔的肿瘤可行胸部B超或CT引导下细针抽吸活检或带芯穿刺活检或小切口开胸手术活检^[2, 3, 6, 8-17]^。中纵隔肿瘤可行纵隔镜活检，或经支气管内镜超声引导活检，或经食管超声引导穿刺活检^[17]^。后纵隔肿瘤可经胸超声或CT引导穿刺活检，但多数情况下需要行VATS手术^[18]^。不同诊断方法的敏感性和特异性见表 1^[1-3, 6, 7, 10-15, 17, 19-22]^。

**1:** 纵隔肿瘤活检诊断的敏感性和特异性^a^ Sensitivity and Specificity of Mediastinal Biopsies for the Diagnosis of Mediastinal Lesions^a^

活检方式	敏感性(%)	特异性(%)	阳性预测值(%)
FNA	71-100	77-100	69-100
EBUS或EUS FNA	38-88	65-100	90-100
带芯穿刺活检	40-93	76-90	83-91
^a^大多数研究报道不同纵隔肿瘤活检的敏感性和特异性，并且认为通过FNA和带芯穿刺活检诊断胸腺瘤是很困难的^[1-3, 6, 7, 10-15, 19-22]^。Zakowski等^[7]^报道，使用FNA诊断胸腺瘤和胸腺癌可以达到100%的准确率。EBUS, 支气管内镜超声；EUS, 食管超声；FNA, 细针抽吸活检；PPV, 阳性预测值。 注：本表已获得版权所有者© 2011 by the International Association for the Study of Lung Cancer复制许可。			

此处不讨论纵隔肿瘤的鉴别诊断，但在活检之前了解患者的年龄、性别、肿瘤位置和影像学表现有助于正确的病理诊断。例如，对于儿童或20岁-30岁的前纵隔肿瘤患者，鉴别诊断要考虑生殖细胞肿瘤、淋巴管瘤、霍奇金淋巴瘤和淋巴母细胞瘤。对于年龄较大的前纵隔肿瘤患者，鉴别诊断则应考虑胸腺瘤或其他胸腺上皮肿瘤^[1]^。发生在上纵隔的肿瘤常为甲状腺起源。而成年人合并重症肌无力的前纵隔肿瘤多为胸腺瘤。中纵隔的肿瘤和囊肿常发生在成年人，多为淋巴瘤、纵隔囊肿、转移瘤、硬化性纵隔炎等。后纵隔肿瘤的鉴别诊断包括神经源性肿瘤、胃肠源性囊肿等^[1]^。纵隔转移瘤最常见的原发部位是肺。常见原发性纵隔肿瘤的好发部位和好发年龄见表 2。

**2:** 常见纵隔肿瘤的好发部位和好发年龄 Common Mediastinal Lesions by Location and Age

项目	纵隔位置	年龄
胸腺瘤	前纵隔	成人
胸腺癌	前纵隔	成人
胸腺增生	前纵隔	成人，儿童
神经内分泌肿瘤	前纵隔	成人
生殖细胞肿瘤	前纵隔	儿童，青年
淋巴母细胞淋巴瘤	前纵隔	儿童，成人
霍奇金淋巴瘤	前纵隔，中纵隔	儿童，成人
弥漫大细胞淋巴瘤	前纵隔	青年
胸腺脂肪瘤	前纵隔	成人
硬化性纵隔炎	前纵隔，中纵隔	成人
Castleman's病	前纵隔，中纵隔	成人
多房性胸腺嚢肿	前纵隔	成人
先天性胸腺嚢肿	前纵隔	儿童，青年
易位甲状腺肿瘤	前纵隔	成人
易位甲状旁腺肿瘤	前纵隔	成人
副神经节瘤	前纵隔	成人
支气管源性嚢肿	中纵隔	成人
心包嚢肿	中纵隔	成人
转移瘤	中纵隔，前纵隔	成人
神经源性肿瘤	后纵隔	儿童，成人
肠源性嚢肿	后纵隔	成人
注：本表得到版权所有者© 2011 by the International Association for the Study of Lung Cancer复制许可。		

## 3 常见活检小标本的获取和病理解释

### 3.1 细针穿刺活检

#### 3.1.1 标本获取的技术要求

关于纵隔活检的操作目前还没有专家共识或循证学证据的规范指南。多数研究报道，使用22G穿刺针的细针抽吸活检能获得较好的结果^[2-7]^。因此，建议细针抽吸活检至少需用22G的穿刺针（表 3）。

**3:** 纵隔肿瘤FNA活检规范 Policies Regarding FNA Biopsies of Mediastinal Lesions

FNA活检要求
22G穿刺针（或更粗）
ROSE或至少3条组织
ROSE或至少6张涂片（每条组织制2张涂片）而且将收集的活检标本保存
在CYTORICH RED或相似的收集液中
若怀疑淋巴瘤，建议行流式细胞仪检查
FNA活检病理报告及说明
病理报告需结合临床和影像学信息^a^
标本是否足够^a^
鉴别诊断应使用免疫组化
当诊断有困难时应咨询其他有经验的病理学家
^a^没有统一的标准，应根据临床能否满足临床鉴别诊断来判断。FNA, 细针抽吸活检；ROSE, 现场实时病理检查。 注：本表得到版权所有者© 2011 by the International Association for the Study of Lung Cancer复制许可。

关于至少需要多少条组织才能获得足够细针抽吸活检样本，和应该制备多少张涂片和切片等问题，目前还没有专家共识和循证医学指南。建议行FNA时由病理学家或细胞技术员对获取的组织进行现场实时病理评估，以确保获得足够的样本用于诊断和研究^[4, 5]^。但是，在临床实践中由于技术人员的短缺和其他的考量很难实现。3条活检组织能够制备足够的涂片用于DiffQuik，Papanicolau染色和各种免疫组化。一般来说，每条活检组织应制备2张以上的涂片，并将剩余的组织浸泡在收集液中（如CYTORICH Red，Termo Fisher Scientifc, Pitsburgh, PA)以制备细胞块。

细针穿刺（fine-needle aspiration, FNA）细胞学标本一般固定在95%的酒精中^[4, 5, 7]^。到底需要多少张涂片也还没有统一的标准。对于大多数患者通常需要现场评估以确保获得足够的标本。如果没有现场评估建议至少准备6张涂片（每条组织2张），多余部分浸泡在如前所述的收集液中或制备细胞块。涂片通常用Diff-Quik、Papanicolau或HE染色，而细胞块切片后可做HE染色、组织化学染色和免疫组化染色。对考虑恶性淋巴瘤的患者，其活检标本应收集在相应的介质中（如Roswell Park Memorial Institute media fluid）以行流式细胞仪检查。

#### 3.1.2 FNA活检的病理解释和报告

关于纵隔FNA活检的报告还没有循证医学证据或专家共识的指南^[4, 5, 7]^。病理医生可选择综观报告或叙述报告，但都应该说明标本量是否足够，见表 3。胸腺瘤FNA活检对获取组织量没有定量标准，不像甲状腺活检可提供细胞或细胞簇数量。细胞学检查应结合临床和影像学表现尽可能给出一个特异性诊断，所以报告中应提供相关的临床信息^[4, 7]^。当鉴别诊断中考虑恶性淋巴瘤可能性大时，细胞学报告中需包含免疫染色和流式细胞仪检查的相关信息^[22]^。免疫组化有助于纵隔肿瘤FNA活检的诊断。免疫标记角蛋白AE1/AE3可有助于判断上皮性肿瘤是否来源于胸腺。免疫标记AFP、HCG、ALP和OCT3/4可有助于鉴别生殖细胞肿瘤与胸腺肿瘤。纵隔肿瘤诊断的各种免疫标记见表 4。当病理医生对纵隔FNA活检诊断经验不足或对特殊诊断结果不确定时，建议与另一位有经验的同事或胸腺瘤病理专家共同阅片。由于很多纵隔肿瘤具有不均一的特征，加之获得的组织量又非常有限，报告FNA活检结果时须非常谨慎。FNA活检通常不能准确诊断胸腺亚型和淋巴瘤的各种亚型，尤其应该避免用FNA活检对胸腺瘤行WHO分类。

**4:** 纵隔肿瘤鉴别诊断的免疫组化标志物选择 Selected immunohistochemical markers used in the differential diagnosis of selected mediastinal lesions

项目	上皮标志物	胸腺癌的混合标志物	神经内分泌标志物	生殖细胞标志物	肺原发标志物	成熟T细胞表型的淋巴样标志物	不成熟表型的淋巴样标志物	淋巴样标志物	
	CK	CD117, CD5^a^, CD70, EMA^a^	Syn, Chromogranin, CD56	OCT3/4, AFP, CD30, PLAP	TTF-1, Napsin, Surfactant Aproprotein	CD3, CD45	CD99, Tdt, CD1a	CD20	
								LY	EC
胸腺瘤	+	-	-	-	-	+	+	-	-/+
胸腺增生	+	-	-	-	-	+	+	+	-
胸腺癌	+	+	+/-	-	-	+	-	-	-
胸腺内分泌肿瘤	+	-	+	-	-	-	-	-	-
淋巴瘤	-	-	-	+(CD30^b^)	-	+	+	+	-
生殖细胞肿瘤	+/-	-	-	+	-	-	-	-	-
转移	+/-	_-_a	+/-	-	+	-	-	-	-
在这些对前纵隔肿瘤评估有帮助的抗体中，一些是评估上皮细胞（EC)成分的，而另一些对淋巴细胞（LY)成分评估有帮助。此外，已证明，一些原来用于诊断血液淋巴细胞的标志物对胸腺上皮肿瘤的诊断是有帮助的，因为它们可以在胸腺瘤或胸腺癌的亚型中异常表达^[26-32]^。^a^胸腺外原发的腺癌常表达CD5和EMA, ^b^纵隔淋巴瘤表达CD30, 霍奇金淋巴瘤和某些纵隔B细胞淋巴瘤中也表达CD30。 注：本表得到版权所有者© 2011 by the International Association for the Study of Lung Cancer复制许可。									

### 3.2 带芯穿刺活检

#### 3.2.1 标本获取的技术要求

19G号Trucut穿刺针常用于带芯穿刺活检^[12, 17, 20]^。18G号的Poin穿刺针在FNA和带芯穿刺中都得到成功应用^[12]^。基于这些结果，我们建议行经胸带芯穿刺活检，至少得用19G穿刺针（表 5）。

**5:** 纵隔肿物带芯穿刺活检规范 Policies regarding needle core biopsies of mediastinal lesions

带芯穿刺活检技术部分
19G穿刺针（或更粗）
3条组织（或更多）
带芯穿刺活检病理说明及报告
病理报告应结合临床和影像学信息
鉴别诊断应使用免疫组化
诊断困难时需咨询其他有经验的病理学家
注：本表得到版权所有者© 2011 by the International Association for the Study of Lung Cancer复制许可。							

关于行带芯经胸穿刺活检需要的最少组织条数目前还没有统一的指南。对于大多数患者至少需要3条组织才能获得足够的标本，而且我们建议将这作为一个标准。带芯穿刺活检组织一般固定在福尔马林中，切片后行HE染色，必要时可以做各种辅助的组织化学染色和免疫组化^[16, 18, 21]^。组织化学和免疫标记的种类和数量根据在活检组织中的病理的发现来决定。

#### 3.2.2 带芯穿刺活检的病理解释和报告

关于纵隔带芯穿刺活检的报告目前还没有循证医学证据和专家共识的指南^[12, 17, 20]^，病理医生可根据喜好选择综观报告或叙述报告。病理医生应紧密结合影像学表现和临床信息对活检作出特异性诊断，而不仅仅是对镜下表现的描述。然而，病理医生也应该意识到，纵隔肿瘤尤其是胸腺瘤存在很大的异质性，故应在报告的评论部分以一种简短的讨论形式向临床医生说明该诊断的潜在局限性。表 4列举了有助于纵隔带芯穿刺活检诊断的免疫标记物。当病理医生对纵隔FNA活检诊断经验不足，或对特殊诊断结果不确定时，建议与另一位有经验的同事或顾问共同阅片。报告带芯穿刺活检的结果时需十分谨慎。带芯穿刺活检通常不能准确区分胸腺瘤亚型和淋巴瘤亚型，因为这些肿瘤存在异质性。所以不能依靠带芯穿刺活检诊断胸腺瘤的亚型。尽管没有带芯穿刺活检诊断胸腺瘤亚型准确性的确切数据，但推测其准确性是有限的，因为需要对切除标本多部位取材检查才能准确诊断^[23-25]^。

### 3.3 手术切除活检

#### 3.3.1 标本获取的技术要求

与细针抽吸活检或带芯穿刺活检相比，手术活检（纵隔切开术，纵隔镜，胸腔镜手术，或小切口开胸术）能为病理医生提供更大的标本^[8]^。但是，关键是要获得足够的有代表性的组织。纵隔肿物外科切取活检常需要送检冰冻病理^[19]^。由于冰冻切片流程中人工技术处理造成的假象，对准确诊断造成一定的阻碍。所以当术中讨论时应提醒外科医生该患者除冰冻切片外尚需要额外的福尔马林或其它方法固定来最终验证诊断（表 6）。此外，对冰冻切片的结果报告要谨慎，尤其涉及到手术决策时^[23]^。

**6:** 纵隔肿瘤手术切取活检规范 Policies regarding surgical incisional biopsies of mediastinal lesions

切取活检要求
冰冻切片判断切取组织是否具有代表性
冰冻切片病理结果应谨慎报告
除冰冻切片外应有足够的组织作常规石蜡切片
因纵隔肿瘤具有不均质特点推荐多点取材
活检强调切取深度，而不是宽度
手术切取活检的病理说明及报告规范
病理报告应结合临床和影像学信息
诊断困难时，建议咨询其他有经验的病理学家
免疫组化有助于区分胸腺恶性肿瘤组织亚型和与其它纵隔肿瘤鉴别
注：本表得到版权所有者© 2011 by the International Association for the Study of Lung Cancer复制许可。

纵隔肿瘤的多点活检在胸腺瘤的正确诊断中起着非常重要的作用。胸腺瘤的形态和亚型在肿瘤内部可呈连续的不均一性，胸腺瘤和胸腺癌可混合存在一个肿瘤中。其它纵隔肿瘤也是如此，如生殖细胞肿瘤和胸腺内分泌肿瘤可混合存在。经多点活检取材可以避免囊性退化区域或坏死区域对诊断的影响。如果有可能尤其怀疑霍奇金淋巴瘤时应取体积为1立方厘米大小的纵隔肿瘤组织送流式细胞术检查，多点活检组织印片也有助于诊断。

#### 3.3.2 切取活检的病理解释和报告

手术活检标本的处理与其他手术病理标本是一样的，但报告仍然建议将活检结果按前面描述的那样与影像学结果紧密联系，当对病理结果不确定时，需要另外一位病理医生重新阅片（表 6）。

表面上，胸腺瘤的诊断似乎很简单，但是，根据外科医生和肿瘤内科医生的考虑，可能需要进行额外的免疫组织化学染色行进一步的鉴别诊断。每一个免疫组化的结果都暗含着特定不同的诊断和相应的外科或内科治疗方案：

   1.胸腺增生与胸腺瘤

   2.胸腺瘤的WHO分型

   3.胸腺瘤与胸腺癌

   4.胸腺瘤与胸腺神经内分泌肿瘤

   5.胸腺癌的亚型，尤其小细胞癌

   6.胸腺上皮肿瘤与转移或其他纵隔原发肿瘤。

免疫组化是解决这些问题的主要方法。如果常规的病理组织不能分类则需要足够的10%的福尔马林固定的诊断组织做免疫组化^[1]^。胸腺瘤是细胞角蛋白阳性的上皮增殖，网状连接的上皮细胞间伴有不同程度的淋巴细胞浸润。这些上皮细胞表达各种上皮抗原，包括CEA、Ber EP4、CD15和钙网膜蛋白。胸腺瘤中的淋巴样成分主要由T细胞构成，很少有B淋巴细胞，并且往往T细胞表型不成熟，表达CD3、CD5、CD43、CD1a、CD99、Tdt、CD10和Bcl2。需要引起注意的是，这些不成熟的表型可与正常胸腺、胸腺增生和淋巴母细胞淋巴瘤相重叠。根据胸腺瘤的上述特点及使用表 4中列举的免疫组化，可以处理这6个主要的问题。

一些临床医生可能根据W HO分类来制定治疗方案。一般来说，A型胸腺瘤可反常表达CD20^[26]^。与其它类型胸腺瘤相比，B3型胸腺瘤可表达EMA、突变的P53蛋白，偶尔也表达CD5。胸腺瘤尤其是B3型胸腺瘤、胸腺神经内分泌肿瘤和淋巴上皮样胸腺癌常常通过形态即可鉴别。但对于一些疑难的病例免疫组化有助于诊断，CD5、CD117（尤其同时表达CD5和CD117）、CD70和突变型P53更常见于胸腺癌。Syn、CD56和嗜铬粒蛋白更常见于神经内分泌癌。代表增殖活性的Ki-67在胸腺癌的表达明显高于胸腺瘤。值得注意的是，淋巴样细胞胸腺瘤Ki-67可表现为强阳性，此时，有必要检查上皮成分与淋巴细胞成分的核分裂像来鉴别胸腺瘤和胸腺癌。另外，器官特异性或神经内分泌染色，如TTF-1，CDX-2，降钙素和PSA等，有助于排除前纵隔类似原发神经内分泌癌或其他胸腺肿瘤的转移瘤。胸腺癌有着广泛的不同组织学类型，一旦诊断为胸腺癌，则需要特异的组织化学和免疫组化染色来对它们进行区分。

胸腺上皮肿瘤可能与恶性淋巴瘤混淆，包括淋巴母细胞淋巴瘤，小淋巴瘤样增生和霍奇金淋巴瘤。细胞角蛋白和CD45染色有助于淋巴造血系统增殖疾病的筛查。在非霍奇金淋巴瘤的分类中，可使用系列的综合性免疫标记物，因此，流式细胞技术是值得做的。对于霍奇金淋巴瘤，CD30、CD15、PAX5和EB病毒的EBER探针有助于鉴别诊断。在年轻患者中需要考虑生殖细胞肿瘤尤其是精原细胞瘤、胚胎癌和卵黄囊瘤。仅20%的精原细胞瘤患者细胞有角蛋白弱表达，而OCT3/4、ALP和podoplanin表达阳性。胚胎癌的细胞角蛋白阳性，但也可表达CD30和CD3/4。卵黄囊瘤细胞角蛋白也呈阳性表达，但同时表达AFP和偶尔表达CD30。前纵隔的间皮瘤和肉瘤鉴别需要特殊的抗体组，不在本文讨论。

或许，最重要的鉴别诊断是其它原发瘤导致的胸腺转移或淋巴结转移，最常考虑的是非小细胞肺癌，TTF-1、Napsin和surfactant apoprotein（表面活性蛋白）染色有助于鉴别诊断。甲状腺癌侵犯纵隔，可以通过甲状腺球蛋白和PAX8免疫染色鉴别。转移性乳腺癌通常GCDFP-15和乳房珠蛋白阳性，以及ER和PR阳性。转移瘤的鉴别诊断中常常要考虑到黑色素瘤，S-100蛋白、HMB45和melan-A免疫过氧化物酶检查有助于诊断。
